# Photocatalytic Degradation of *p*-Cresol by Zinc Oxide under UV Irradiation

**DOI:** 10.3390/ijms13010302

**Published:** 2011-12-27

**Authors:** Yadollah Abdollahi, Abdul Halim Abdullah, Zulkarnain Zainal, Nor Azah Yusof

**Affiliations:** 1 Advanced Materials and Nanotechnology Laboratory, Institute of Advanced Technology, Universiti Putra Malaysia, 43400 Serdang, Selangor D.E., Malaysia; E-Mails: zulkar@science.upm.edu.my (Z.Z.); 2 Department of Chemistry, Faculty of Science, Universiti Putra Malaysia, 43400 UPM Serdang, Selangor, Malaysia; E-Mail: azah@science.upm.edu.my

**Keywords:** photocatalytic degradation, *p*-cresol, mineralization, ZnO, UV

## Abstract

Photocatalytic degradation of *p*-cresol was carried out using ZnO under UV irradiation. The amount of photocatalyst, concentration of *p*-cresol and pH were studied as variables. The residual concentration and mineralization of *p*-cresol was monitored using a UV-visible spectrophotometer and total organic carbon (TOC) analyzer, respectively. The intermediates were detected by ultra high pressure liquid chromatography (UPLC). The highest photodegradation of p-cresol was observed at 2.5 g/L of ZnO and 100 ppm of p-cresol. *P*-cresol photocatalytic degradation was favorable in the pH range of 6–9. The detected intermediates were 4-hydroxy-benzaldehyde and 4-methyl-1,2-benzodiol. TOC studies show that 93% of total organic carbon was removed from solution during irradiation time. Reusability shows no significant reduction in photocatalytic performance in photodegrading *p*-cresol.

## 1. Introduction

Photocatalysis is a subject of interest in view of its prosperous application in pollutant decontamination. Photocatalysis takes the advantage of the ability of semiconductor photocatalyst to generate surface bound hydroxyl radical and trapped hole upon excitation by band gap light [1–3]. Basically, under illumination by suitable light, this process (Equations 1–8) produces hydroxyl radical and hole which are powerful oxidants that can degrade a variety of organic compounds [1,4,5].

(1) $$\text{Photoexcitation }:\text{photocatalyst}+\text{h}\nu \to {\text{e}}^{-}+{\text{h}}^{+}$$

(2) $$\text{Adsorbed oxygen}:{\left({\text{O}}_{2}\right)}_{\text{ads}}+{\text{e}}^{-}\to {{\text{O}}_{2}}^{-•}$$

(3) $$\text{Ionization of water}:{\text{H}}_{2}\text{O}\to {\text{OH}}^{-}+{\text{H}}^{+}$$

(4) $$\text{Protonation of superoxides}:{{\text{O}}_{2}}^{-•}+{\text{H}}^{+}\to {\text{HOO}}^{•}$$

(5) $${\text{HOO}}^{•}+{\text{e}}^{-}\to {{\text{HO}}_{2}}^{-}$$

(6) $${\text{HOO}}^{-}+{\text{H}}^{+}\to {\text{H}}_{2}{\text{O}}_{2}$$

(7) $${\text{H}}_{2}{\text{O}}_{2}+{\text{e}}^{-}\to {\text{OH}}^{-}+{\text{OH}}^{•}$$

(8) $${\text{H}}_{2}\text{O}+{\text{h}}^{+}\to {\text{H}}^{+}+{\text{OH}}^{•}$$

Among the several semiconductor photocatalysts used, TiO_2_ has been considered the most superior in terms of suitability for application [6,7]. The current interest in ZnO is based on its high spectral response in UV region, which presumably, in some studies, resulted in higher efficiency of photocatalytic degradation well over TiO_2_ [8–12]. Based on the aforementioned, ZnO photocatalysis has been proposed as an alternative in the removal of various aqueous pollutants including phenolic compounds [13–19]. *P*-cresol as a phenolic compound has been listed as the priority [20]. Water solubility of *p*-cresol is above 21.5 g/L (25 °C) [21]. Therefore, *p*-cresol can be a significant threat to surface water, groundwater sources, or generally the environment [7,22].The effective removal of *p*-cresol is currently an environmental problem [23,24]. In our previous works, the effect of operational parameters on photocatalytic degradation of *m*-cresol [25] and *o*-cresol [26] was reported by UV and visible/ZnO process. However, no study has been conducted on aquatic *p*-cresol photocatalytic degradation using ZnO under UV irradiation. We undertake to investigate the effect of operating parameters such as *p*-cresol concentration, amount of photocatalyst and pH on degradation efficiency. In addition, the mineralization and photoproducts were investigated by total organic carbon (TOC) measurement and Waters-Acquity ultra high pressure liquid chromatography (UPLC).

## 2. Materials and Methods

**Figure 1:**
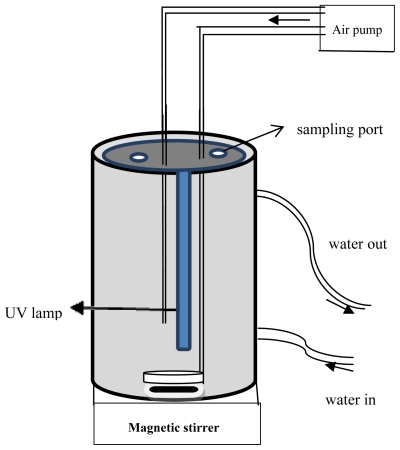
Schematic diagram of batch UV photoreactor.

*P*-cresol (99.5%, Fluka), NaOH (99% Merck), H_2_SO_4_ (95%–97%) and other required chemicals were of reagent grade, obtained from Merck and were used without further purification. The ZnO (99%, Merck) has a surface area of 3.3 m^2^/g measured by static BET using Thermo Finnigan Sorptomatic 1990 Series analyzer. The particle size of ZnO recorded on Nanophox facility was 0.4–0.5 μm. Band gap measured using PerkinElmer Lambda 35 UV/vis/NIR was 3.02 eV. In all photocatalytic experiments, a litter of mixture ZnO with known quantities and *p*-cresol was irradiated for 360 min. Photocatalytic experiments were performed in a non-continuous mode (batch) binary reactor (see Figure 1) fitted with 6W UV-A lamp. The light source has maximum intensity at 365 nm. The mixture was magnetically stirred (200 rpm) to maintain even distribution of suspension throughout the reactor and eliminated mass gradient. To make the produced gas volatile (CO_2_), increase solution fluidization and access oxygen for Equation (2), air was blown into the reaction solution using an air pump at a flow rate of 150 L/h. Flowing cooled water into the binary cylinder for keeping the temperature at around 25 °C. At specific time intervals, samples were withdrawn from the bulk solution. The samples were filtered through a 0.45 μm polytetrafluro-ethylene (PTFE) membrane. In order to compare the efficiency of the photocatalytic degradation of *p*-cresol, the filtrates were analysed by UV-Visible spectrometry (Shimadzu, UV-1650pc) at the maximum absorption wavelength of *p*-cresol (277 nm). The intermediates were identified by UPLC. The used UPLC was fitted with an Acquity BEH phenyl C18 column (10 cm × 2.1 mm × 1.7 μm) and the detector wavelength was 271 nm. The gradient elution method was applied over a 3-minute run time. The mobile phase was acetonitrile (65%)-water (35%), while a photodiode array (PDA) spectrometer operated at fixed detection wavelength for each experiment was used as a detector. Mineralization was measured by TOCVCSN analyzer. The percentage photocatalytic degradation of *p*-cresol was calculated using Equation (9).

(9) $$\text{Photodegradation}\%=100\times [(C_{0}-C_{\text{t}})/C_{0}]$$

where *C*_0_ = initial concentration of *p*-cresol, *C*_t_ = concentration of *p*-cresol after photoirradiation. All photocatalytic degradation experiments were carried out in duplicate.

Photocatalytic degradation of *p*-cresol was investigated in the absence of photocatalyst and at normal pH (7.49). Results show only 6% of *p*-cresol was photolysed in the absence of photocatalyst. Therefore, *p*-cresol is relatively stable under UV irradiation. The concentration of *p*-cresol was determined in the presence of photocatalyst in the dark. 7% of *p*-cresol reduction was observed base on that it is suggested that the adsorption takes place on the photocatalyst surface [27].

## 3. Results and Discussion

### 3.1. Effect of Photocatalyst Loading

**Figure 2:**
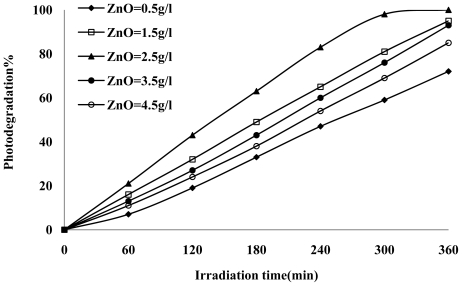
Effect of photocatalyst loading on photodegradation of *p*-cresol under UV, initial condition: *p*-cresol concentration = 100 ppm and pH = 7.49.

A series of experiments were carried out by varying the amount of catalyst (0.5 to 4.5 g/L) to establish the effect of photocatalyst loading and to avoid unnecessary excess photocatalyst. The percentage of photodegraded *p*-cresol as a function of irradiation time was plotted in Figure 2. The maximum percentage of photodegradation was obtained at 2.5 g/L of photocatalyst. This is due to the increase in number of active sites with increasing photocatalyst loading which consequently leads to enhanced production of ^•^OH radicals. Moreover, the number of adsorbed *p*-cresol molecules increased with the increase in the number of photocatalyst particles, thus increase the percentage of photodegradation [27]. When the amount of photocatalyst was increased beyond optimum, the percentage degradation decreased. This can be attributed to the increase in the turbidity of the solution that reduces the light penetration through the solution known as light screening effect [7]. Furthermore, agglomeration and sedimentation of photocatalyst particles is also possible [28]. In such condition, a part of the photocatalyst surface probably becomes unavailable for photon absorption and *p*-cresol adsorption, thus reduces the photocatalytic reaction.

### 3.2. Effect of p-Cresol Concentration

**Figure 3:**
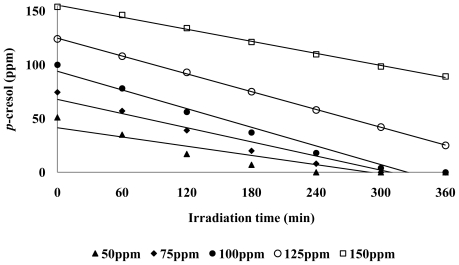
Effect of *p*-cresol concentration on photocatalytic degradation under UV irradiation, initial condition: amount of photocatalyst = 2.5g/L and pH = 7.49.

The photocatalytic degradation of various *p*-cresol concentrations was studied. The results (Figure 3) showed that the percentage degradation decreased with increasing *p*-cresol concentration. At a high *p-*cresol concentration (150 ppm), the presumption is that the active sites are covered by *p*-cresol and its intermediates that can cause reduced generation of electron-hole pair (e^−^-h^+^), which subsequently reduces the photodegradation efficiency [28]. Since the mass of photocatalyst, the intensity of light and illumination time were kept constant, as the initial *p*-cresol concentration increased, the ^•^OH and O_2_^−•^ species formed on the surface of photocatalyst would therefore remain constant. Thus the relative ratio of the ^•^OH and O_2_^−•^ for attacking *p*-cresol decreased which led to a reduction in percentage degradation of p-cresol[29]. Another factor which may be responsible for the reduction in photocatalytic degradation rate is the competition between adsorbed *p*-cresol and H_2_O molecules for photodegraded h^+^ [25].

### 3.3. Photodecomposition Kinetics

**Figure 4:**
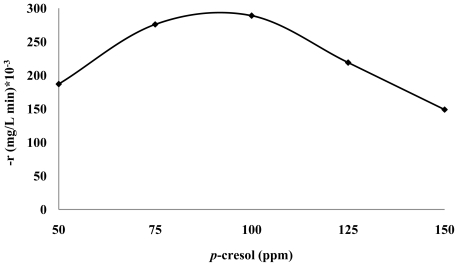
Rate constants (r) of photodegradation of different *p*-cresol concentrations under UV irradiation. Initial condition: ZnO = 2.5g/L, pH = 7.49, at 25 °C (Derived from Figure 3).

The rate of the photocatalytic degradation *vs. p*-cresol concentration exhibited a quadratic behaviour which has an optimum value at 100 ppm (Figure 4). The rate is a nonlinear function of *p*-cresol concentration (*C*_cresol_). The reason for this behavior may be related to the probability of interaction between *p*-cresol and ZnO surface. At very low *p*-cresol concentration, the probability of interaction between *p*-cresol molecules and surface of ZnO decreases. Hence, photodegradation rate decreases. The probability of interaction between substrates molecules and oxidizing species is increased by increasing *p*-cresol concentration, leading to an enhancement in the degradation rate [29–31]. Therefore, according to Langmuir-Hinshelwood’s modeling [32], the suggested model is:

(10) $$(-r)=\frac{k_{\text{cresol}}C_{\text{cresol}}}{{\left[1+k_{\text{cresol}}{\text{C}}_{\text{cresol}}\right]}^{2}}$$

where, the estimated value of *k*_cresol_ (mg L^−1^ min^−1^ ppm^−1^) and *K*_cresol_ (ppm^−1^) were 0.0403 and 0.33 with R-squared value 0.98 respectively. Similar results were reported for photodegradation kinetics of aqueous sodium oxalate, sodium dodecyl sulfate, 4-nitrophenol solution using TiO_2_ photocatalyst [33–36] and our previous work [25].

### 3.4. Effect of pH

It is important to study the effect of pH in the photodegradation of *p*-cresol because the charge of ZnO surface and *p*-cresol vary with shift in pH. Primarily, ZnO will be hydroxylated in the presence of water to form hydroxide layers (Zn-OH) [37]. The zinc hydroxide surface (Zn-OH) can become charged by reacting with H^+^ (acidic environment) or OH^−^ (basic environment) ions due to surface amphoteric reactions (Equations 11,12) [38].

(11) $$\text{Zn}-\text{OH}+{\text{H}}^{+}\to \text{Zn}-{{\text{OH}}_{2}}^{+}(\text{acidic environment})$$

(12) $$\text{Zn}-{\text{OH}+\text{OH}}^{-}\to \text{Zn}-{\text{O}}^{-}{+\text{H}}_{2}\text{O }(\text{basic environment})$$

At low pH hydroxide surfaces adsorb protons to produce positively charged surfaces. At high pH (usually above pH 9) they lose protons to produce negatively charged surfaces [39]. The zero point charge of ZnO (pH_zpc_) has been reported to be 9 [40]. Therefore, the surface functional groups of ZnO can be ZnOH_2_^+^, ZnOH, and ZnO^−^ at pH < pH_zpc_, pH_zpc_ and pH > pH_zpc_, respectively.

On the other hand, since *p*-cresol is a strong base (p*K*_a_ ≈ 10), in acidic conditions, more *p*-cresol molecules would tend to be positively charged while the lone pair of electrons of the OH group will be more available for hydrogen bonding (undissociated *p*-cresol) [41]. At high pH (pH ≥ pK_a_), *p*-cresol would exist as a negatively charged 4-methylphenolate species (Equation 13).

(13)
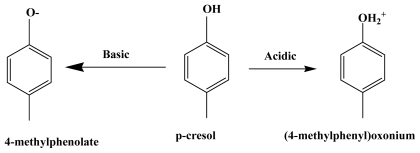


**Figure 5:**
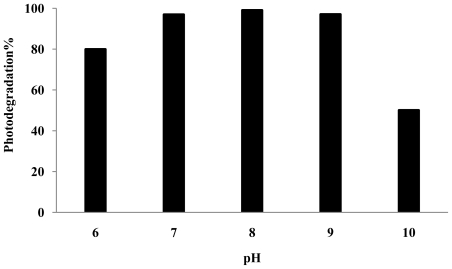
Photocatalytic degradation of *p*-cresol at variation initial pH under UV irradiation, initial *p*-cresol concentrations = 100 ppm, amount of photocatalyst = 2.5 g/L, end of 6 h.

**Figure 6:**
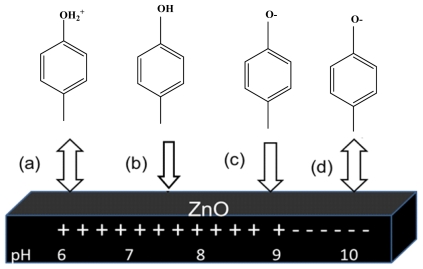
Schematic view of ZnO surface and *p*-cresol in different solution acidity. Several events are illustrated: (**a**) strong repulsion; (**b**) interaction; (**c**) strong attraction (**d**) strong repulsion.

The optimum condition was used as in the previous experiment. As observed, the percentage photodegradation of *p*-cresol increased slightly with increasing pH from pH 6 to 9 (Figure 5). The increase in photodegradation percentage may be due to an increase adsorption of *p*-cresol on the ZnO surface (Figure 6).

It has been reported that in a slightly alkaline solution (pH 8–9), hydroxyl radicals are more easily generated by oxidizing the available OH^−^ on the photocatalyst surface [42]. Thus, generally, the photodegradation efficiency is expected to be enhanced with increasing pH due to the ready availability of hydroxyl radicals for the reaction. However, a decrease in photodegradation percentage was observed at pH 10 (Figure 5). This can be attributed to the reduction for *p*-cresol adsorbed on the catalyst surface at pH 10 (Figure 6). It should also be noted that hydroxyl radicals are rapidly scavenged in presence of excess concentrations of hydroxyl ions and therefore would not have the opportunity to react with the substrates [43]. Hence, a drastic drop in the amount of *p*-cresol photodegraded was observed at pH 10.

### 3.5. Mineralization

The details of the mechanism of *p*-cresol degradation in the presence of hydroxyl radical (^•^OH) until mineralization have been reported in a previous study with boron-doped diamond [44]. It is well known that aromatic intermediates are formed from phenolic compounds, which result thereafter in the formation of carboxylic acids. These carboxylic acids are easily transformed into CO_2_ and H_2_O. The CO_2_ is volatized as gas from the solution [23,45].

**Figure 7:**
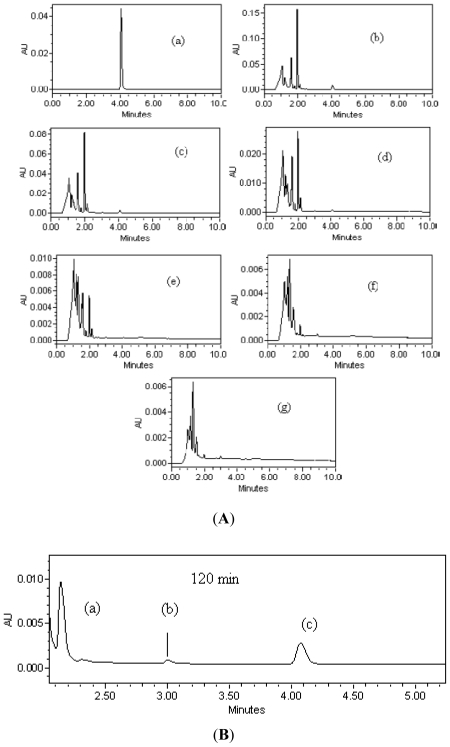
(**A**) *P*-cresol ultra high pressure liquid chromatography (UPLC) chromatograms depicting eluted peaks at different reaction times: (**a**) 0 min; (**b**) 60 min; (**c**) 120 min; (**d**) 180 min; (**e**) 240 min; (**f**) 300 min; (**g**) 360 min., initial condition: *p*-cresol concentration = 50 ppm; amount of ZnO = 2.5 g/L and pH 7.49. (**B**) Selected UPLC chromatograms of *p*-cresol that shows: (a) 4-hydroxy-benzaldehyde; (b) 4-methyl-1,2-benzodiol as intermediates; and (c) *p*-cresol. Concentration of *p*-cresol = 50 ppm at 120 min.

**Figure 8:**
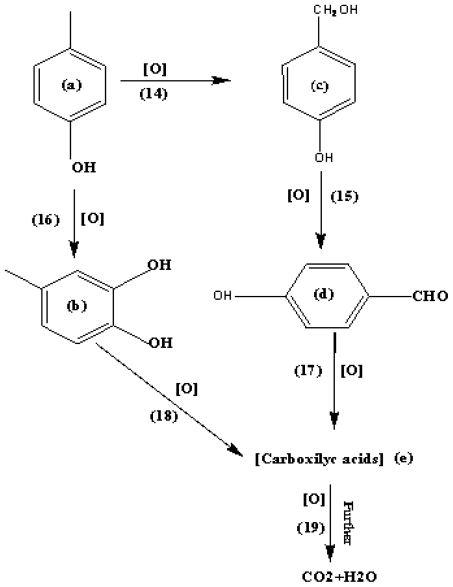
Reaction scheme proposed to account for the formation of degradation intermediates of *p*-cresol on illuminated aqueous ZnO: (a) *p*-cresol; (b) 4-methyl-1,2-benzodiol; (c) undetected; (d) 4-hydroxy-benzaldehyde; (e) carboxylic acids. [O] = HO•, O_2_^−•^, H_2_O_2_ and h^+^

Some of the photoproducts of photodegraded *p*-cresol were determined using UPLC (Figure 7A). The chromatograms show many peaks which may be due to unidentified aromatic intermediates [23]. In this study, the intermediates detected during 120 min of reaction are 4-hydroxy-benzaldehyde (0.2 ppm) and 4-methyl-1,2-benzodiol (0.01 ppm) with retention time (*R*_t_) = 2.145 and *R*_t_ = 3.005 min respectively (Figure 7B). Both intermediates were reported earlier [45]. The ability of semiconductor photocatalysts to remove pollutants is based on the active oxidizing species (HO^•^, O_2_^−•^, H_2_O_2_ and .h^+^) which are produced by the irradiation of the semiconductor (Equations 1–8). The hole is produced as a result of photoinduced charge separation which may then cause the formation of hydroxyl radical by directing the hole transfer reaction with ^−^OH and H_2_O. Hydrogen peroxide may be generated via chain reactions involving conduction band electron. Even though hole and hydrogen peroxide may aid the degradation of *p*-cresol on ZnO dispersion, experimental evidences have proved the main oxidant to be hydroxyl radical [46,47]. Therefore, we propose the mechanistic pathways shown in Figure 8 to account for the photoproducts of *p*-cresol degradation encountered in this study based on hydroxyl radical intervention. Thus, 4-hydroxy-benzaldehyde and 4-methyl-1,2-benzenediol can be produced by through reactions shown by Equation 14–15 and Equation 16, respectively. It is believed that these intermediates are on the brink of ring opening [23]. Thereafter carboxylic acids are formed (Equations 17 and 18). The chain of carboxylic acids is decreased with increasing irradiation time. The final carboxylic acid by product is oxalic acid (HOOC-COOH) which is easily transformed to CO_2_ and H_2_O (Equation 19) by taking two hydroxyl radicals.

**Figure 9:**
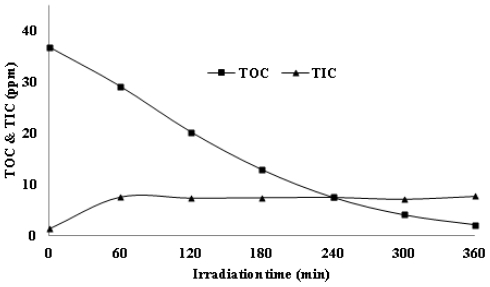
The amount of total organic carbon (TOC) and total inorganic carbon (TIC) during photocatalic degradation of *p*-cresol under UV irradiation, initial condition; *p*-cresol concentration = 50 ppm, ZnO = 2.5 g/L and pH = 7.49.

Mineralization, the main aim of photodegradation of *p*-cresol, was followed by measuring the total organic carbon (TOC) [48] and total inorganic carbon (TIC). Figure 9 shows the TOC and TIC values of *p*-cresol. The amount of TOC steadily decreased with increasing irradiation time, which indicates the decline of *p*-cresol (or intermediates) when irradiation time is increased. On the other hand, the TIC curve shows that the amount of total inorganic carbon was generated in the first hour of reaction and was constant thereafter. This is most likely due to some organic carbon converting into inorganic carbon, for example, carbonate ions, and after a few minutes TIC remained constant. Based on the results, it can be concluded that 93% organic carbon is removed from *p*-cresol solution as CO_2_. The residual TOC value (7%) indicates the presence of other photoproducts such as carboxylic acids at the end of the reaction.

### 3.6. Reusability

**Figure 10:**
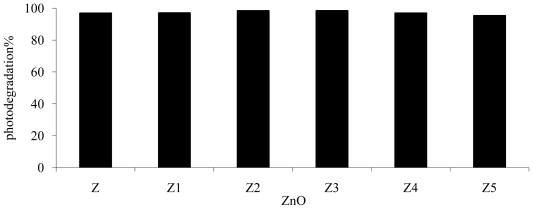
Reusability of ZnO in photodegrading *p*-cresol solution under UV. Z is fresh ZnO cycle and Z1, Z2, Z3, Z4 and Z5 are reused ZnO cycle. Initial condition; concentration of *p*-cresol = 100 ppm, ZnO concentration = 2.5 g/L, pH = 7.49, irradiation time = 6 h.

The reusability of photocatalyst was investigated in order to establish the stability (Figure 10) while studying reuse of photocatalyst; all parameters including irradiation time, pH, *p*-cresol concentration, amount of photocatalyst and irradiation time were kept constant. The photocatalyst was separated from the solution mixture through filtration. The recovered photocatalyst was washed five times with deionized water, dried at 96 °C in oven and reused five times as in the previous degradation process. Results show no significant reduction in photocatalytic performance in photodegrading *p*-cresol, thus this indicates the stability of ZnO as a photocatalyst. Moreover, inductively coupled plasma (ICP) showed that the photocorrosion of ZnO is quite insignificant.

## 4. Conclusion

The *p*-cresol can be photocatalytically degraded using ZnO as photocatalyst under UV irradiation. The efficiency of the photodegradation process is affected by the amount of photocatalyst, concentration of *p*-cresol and the initial pH of the solution. Under optimum conditions, 100 ppm of *p-*cresol can be effectively photodegraded by 2.5 g/L of ZnO. *P*-cresol photodegradation was favorable in the range pH 6–9. The detected intermediates are 4-hydroxy-benzaldehyde, 4-methyl-1,2-benzodiol, as for *p*-cresol. TOC studies show that 93% of total organic carbon is removed from the solution during irradiation time. Reusability shows no significant reduction in photocatalytic performance in the photodegradation of *p*-cresol. This study indicates the great potential of ZnO to remove aqueous *p-*cresol under UV irradiation.
